# Supplementary material for: Hybrid longitudinal-transverse phonon polaritons
Source: Nat Commun. 2019 Apr 11;10:1682. doi: 10.1038/s41467-019-09414-4 (PMC6459832; doi:10.1038/s41467-019-09414-4)
Supplement: Supplementary file 1 — Supplementary Information [file 41467_2019_9414_MOESM1_ESM.pdf]

Supplementary Note: Hybrid Longitudinal-Transverse Phonon Polaritons

Christopher R. Gubbin,[1] Rodrigo Berte,[2,3] Michael A. Meeker,[4] Alexander J. Giles,[4] Chase T. Ellis,[4] Joseph G. Tischler,[4] Virginia D. Wheeler,[4] Stefan A. Maier,[3] Joshua D. Caldwell,[5] and Simone De Liberato[1,*]

[1]*School of Physics and Astronomy, University of Southampton, Southampton, SO17 1BJ, United Kingdom*
[2]*CAPES Foundation, Ministry of Education of Brazil, Brasilia, DF 70040-020, Brazil*
[3]*Chair in Hybrid Nanosystems, Nanoinstitute Munich, Faculty of Physics, Ludwig-Maximilians-Universität München, 80539 München*
[4]*U.S. Naval Research Laboratory, Washington, DC 20375 United States*
[5]*Department of Mechanical Engineering, Vanderbilt University, Nashville, Tennessee 37205 United States*

Supplementary Note 1: Formalism

We can write the ionic equation of motion for a polar dielectric crystal coupled to an electric field, in the form [1–3]

$$\left[\omega_{\mathrm{T}}^2 - \omega(\omega + i\gamma)\right]\mathbf{X} = -\beta_{\mathrm{L}}^2\nabla(\nabla\cdot\mathbf{X}) + \beta_{\mathrm{T}}^2\nabla\times\nabla\times\mathbf{X} - \frac{\alpha}{\rho}(\nabla\phi - i\omega\mathbf{A}), \tag{1}$$

where ω_{T} is the polar dielectric's transverse phonon frequency, γ the phonon damping rate, $\mathbf{X}$ is the ionic displacement, α is the light-matter coupling strength given by

$$\alpha = \sqrt{\rho\epsilon_0\epsilon_\infty\left(\omega_{\mathrm{L}}^2 - \omega_{\mathrm{T}}^2\right)}, \tag{2}$$

ρ is the density, β_{T} (β_{L}) the transverse (longitudinal) phonon velocities and ϕ ($\mathbf{A}$) is the scalar (vector) potential related to the electromagnetic fields $\mathbf{E}, \mathbf{H}$ by

$$\begin{aligned}\mathbf{E} &= -\nabla\phi + i\omega\mathbf{A},\\ \mathbf{H} &= \frac{1}{\mu_0}\nabla\times\mathbf{A}.\end{aligned} \tag{3}$$

Taking the divergence (curl) of Eq. 1 and defining auxiliary scalar (vector) potentials $Y = \nabla\cdot\mathbf{X}$, ($\mathbf{\Theta} = \nabla\times\mathbf{X}$) we can find decoupled equations of motion for the longitudinal auxiliary potential

$$\left[(\omega_{\mathrm{L}}^2 - \omega(\omega+i\gamma)) + \beta_{\mathrm{L}}^2\nabla^2\right]Y = 0, \tag{4}$$

and transverse one

$$\left[(\omega_{\mathrm{T}}^2 - \omega(\omega+i\gamma)) + \beta_{\mathrm{T}}^2\nabla^2\right]\mathbf{\Theta} = i\omega\frac{\alpha}{\rho}\nabla\times\mathbf{A}, \tag{5}$$

where we utilised the Coulomb gauge condition $\nabla\cdot\mathbf{A} = 0$, the absence of free charges $\nabla\cdot\mathbf{D} = 0$, and the constitutive relation linking the electric and displacement fields to the ionic displacement

$$\mathbf{D} = \epsilon_0\epsilon_\infty\mathbf{E} + \alpha\mathbf{X}. \tag{6}$$

*Corresponding author: s.de-liberato@soton.ac.uk

We also introduced the longitudinal phonon frequency

$$\omega_{\mathrm{L}}^2 = \omega_{\mathrm{T}}^2 + \frac{\alpha^2}{\epsilon_0\epsilon_\infty\rho}, \tag{7}$$

where ϵ_∞ is the high-frequency dielectric constant of the polar dielectric. In terms of the newly defined potentials we can reconstruct the ionic displacement by substitution as

$$\mathbf{X} = \frac{1}{\omega_{\mathrm{T}}^2 - \omega(\omega+i\gamma)}\left[\beta_{\mathrm{T}}^2\nabla\times\mathbf{\Theta} - \frac{\beta_{\mathrm{L}}^2\epsilon_\infty}{\epsilon(\omega,0)}\nabla Y - \frac{\alpha}{\rho}(\nabla\phi_{\mathrm{H}} - i\omega\mathbf{A})\right], \tag{8}$$

where we recognised the dielectric function of a polar dielectric crystal in the absence of spatial dispersion

$$\epsilon(\omega,0) = \epsilon_\infty\frac{\omega_{\mathrm{L}}^2 - \omega(\omega+i\gamma)}{\omega_{\mathrm{T}}^2 - \omega(\omega+i\gamma)}. \tag{9}$$

Longitudinal Equation

The longitudinal equation of motion Eq. 4 is just a scalar Helmholtz equation. Taking the divergence of the displacement field $\mathbf{D}$ as defined in Eq. 6 we can find

$$\nabla^2\phi = \frac{\alpha}{\epsilon_0\epsilon_\infty}Y, \tag{10}$$

whose solution is simply given by

$$\phi = \phi_{\mathrm{H}} - \frac{\alpha}{\epsilon_0\epsilon_\infty}\frac{\beta_{\mathrm{L}}^2}{\omega_{\mathrm{L}}^2 - \omega(\omega+i\gamma)}Y, \tag{11}$$

where ϕ_{H} is the homogeneous solution of Eq. 10 satisfying $\nabla^2\phi_{\mathrm{H}} = 0$.

Transverse Equation

Using Maxwell's curl equation in conjunction with the constitutive relation in Eq. 6

$$\nabla\times\mathbf{H} = \frac{\partial\mathbf{D}}{\partial t} = \epsilon_0\epsilon_\infty\frac{\partial\mathbf{E}}{\partial t} + \alpha\frac{\partial\mathbf{X}}{\partial t}, \tag{12}$$

we can put Eq. 5 for the transverse potential in the form

$$i\mu_0\alpha\omega\mathbf{\Theta} = \left[\frac{\omega^2\epsilon_\infty}{c^2} + \nabla^2\right]\nabla\times\mathbf{A}. \tag{13}$$

Making a spatial Fourier transform $\nabla^2 \to -k^2$ and substituting this back into the transverse equation of motion we arrive at the result

$$\left[k^2 - \frac{\omega^2}{c^2}\epsilon(\omega, k)\right]\mathbf{\Theta} = 0, \tag{14}$$

where the easily identified spatially dispersive dielectric function is given by

$$\epsilon(\omega, \mathbf{k}) = \epsilon_\infty \frac{\omega_\mathrm{L}^2 - \omega(\omega + i\gamma) - \beta_\mathrm{T}^2 k^2}{\omega_\mathrm{T}^2 - \omega(\omega + i\gamma) - \beta_\mathrm{T}^2 k^2}, \tag{15}$$

which reduces to the non-dispersive Eq. 9 in the limit $\beta_\mathrm{T} k \ll \omega$.

Supplementary Note 2: Application to a Polar Halfspace

Here we apply the general theory previously derived to the specific case of an a-cut polar dielectric halfspace occupying $z < 0$ whose c-axis is aligned with the x-axis. The region $z > 0$ is filled with a non-resonant dielectric whose dispersionless dielectric function is given by ϵ_B. The system is illuminated from $z = \infty$ by a TM polarised electromagnetic field with incident wavevector in the xz plane.

As we saw in the general theory, solutions to the general isotropic equation of motion in the lower halfspace Eq. 1 are separable into three classes. Firstly the homogeneous solution, satisfying $\nabla^2\phi_\mathrm{H} = 0$ whose solution is given by

$$\phi_\mathrm{H} = -\frac{t_\mathrm{H}}{\epsilon_0\omega} e^{ik_x x} e^{k_x z}, \tag{16}$$

yields the following electric and displacement fields in the lower halfspace

$$\begin{aligned}\mathbf{E}_\mathrm{H} &= -\nabla\phi_\mathrm{H} = \frac{t_\mathrm{H} k_x}{\omega\epsilon_0}(i\hat{\mathbf{x}} + \hat{\mathbf{z}})e^{ik_x x + k_x z},\\ \mathbf{X}_\mathrm{H} &= \frac{\alpha}{\rho\epsilon_0\omega}\frac{t_\mathrm{H} k_x}{\omega_\mathrm{T}^2 - \omega(\omega + i\gamma)}(i\hat{\mathbf{x}} + \hat{\mathbf{z}})e^{ik_x x + k_x z},\end{aligned} \tag{17}$$

which are chosen to ensure decay away from the interface as $z \to -\infty$. Here t_H is a constant to be determined by application of the electromagnetic and mechanical boundary conditions. In the upper halfspace where $\alpha = 0$ it is clear that the scalar equation Eq. 10 still admits a homogeneous solution whose out-of-plane wavevector is equal and opposite to that in the lower halfspace. Considering this mode allows the homogeneous solution to be ignored in calculation of the electromagnetic boundary conditions.

Zone-centre longitudinal modes are not considered as solutions of Eq. 4 due to their far off-resonant nature. As a result of Bragg scattering along the crystal c-axis, which for the a-cut system considered is parallel to the x-axis, the in-plane wavevector of the Bragg scattered modes is shifted as $k_{x\mathrm{L}} = k_\mathrm{M} + k_x$ in which $k_\mathrm{M} = \pi/a$ where a is the lattice constant along the c-axis. The scalar potential is therefore given by

$$Y = \frac{\alpha\mu_0 t_\mathrm{L}}{\rho\omega} e^{ik_{x\mathrm{L}}x + ik_{z\mathrm{L}}z}, \tag{18}$$

in which t_L is a constant to be determined by application of the appropriate boundary conditions. These modes are subject to the dispersion relation

$$0 = \omega_\mathrm{L}^2 - \omega(\omega + i\gamma) - \beta_\mathrm{L}^2 k_{x\mathrm{L}}^2 - \beta_\mathrm{L}^2 k_{z\mathrm{L}}^2, \tag{19}$$

which yields the out-of-plane longitudinal wavevector $k_{z\mathrm{L}}$.

The corresponding electric potential is calculated through the inhomogeneous component of Eq. 11 as

$$\phi_\mathrm{L} = -\frac{\alpha^2}{\rho\epsilon_0^2\epsilon_\infty\omega}\frac{\beta_\mathrm{L}^2/c^2}{\omega_\mathrm{L}^2 - \omega(\omega + i\gamma)} t_\mathrm{L} e^{ik_{x\mathrm{L}}x} e^{ik_{z\mathrm{L}}z}, \tag{20}$$

generating the electric field

$$\begin{aligned}\mathbf{E}_\mathrm{L} = -\nabla\phi_\mathrm{L}, &= \frac{i\beta_\mathrm{L}^2 t_\mathrm{L}}{c^2\epsilon_0\omega}\left[1 - \frac{\epsilon_\infty}{\epsilon(\omega, 0)}\right]\\ &\times\left[k_{x\mathrm{L}}\hat{\mathbf{x}} + k_{z\mathrm{L}}\hat{\mathbf{z}}\right] e^{ik_{x\mathrm{L}}x} e^{ik_{z\mathrm{L}}z},\end{aligned} \tag{21}$$

and ionic displacement through Eq. 8

$$\begin{aligned}\mathbf{X}_\mathrm{L} = &-\frac{\beta_\mathrm{L}^2}{\omega_\mathrm{L}^2 - \omega(\omega + i\gamma)}\nabla Y = -\frac{i\alpha t_\mathrm{L}}{\rho\epsilon_0\omega}\frac{\beta_\mathrm{L}^2/c^2}{\omega_\mathrm{L}^2 - \omega(\omega + i\gamma)}\\ &\times\left[k_{x\mathrm{L}}\hat{\mathbf{x}} + k_{z\mathrm{L}}\hat{\mathbf{z}}\right] e^{ik_{x\mathrm{L}}x + ik_{z\mathrm{L}}z}.\end{aligned} \tag{22}$$

Finally as a solution to Eq. 5 we consider a TM polarised transverse field, whose magnetic component is given by

$$\mathbf{H} = t_\mathrm{T} e^{ik_x x} e^{ik_{z\mathrm{T}}z}\hat{\mathbf{y}}, \tag{23}$$

where t_T is a constant to be determined by applying the electromagnetic and mechanical boundary conditions and $k_{z\mathrm{T}}$ is the out-of-plane wavevector for the transverse mode. This mode is generated by the vector potential

$$\mathbf{A} = \frac{t_\mathrm{T}}{\omega^2\epsilon_0\epsilon(\omega, \mathbf{k}_\mathrm{T})}\left[-ik_{z\mathrm{T}}\hat{\mathbf{x}} + ik_x\hat{\mathbf{z}}\right] e^{ik_x x} e^{ik_{z\mathrm{T}}z}, \tag{24}$$

from which we can calculate the auxiliary vector potential

$$\mathbf{\Theta} = \frac{i}{\alpha\omega}\frac{\omega^2}{c^2}\left[\epsilon(\omega, \mathbf{k}_\mathrm{T}) - \epsilon_\infty\right] t_\mathrm{T} e^{ik_x x} e^{ik_{z\mathrm{T}}z}\hat{\mathbf{y}}. \tag{25}$$

Now we are in a position to calculate the transverse electric field

$$\mathbf{E}_\mathrm{T} = i\omega\mathbf{A} = \frac{t_\mathrm{T}}{\omega\epsilon_0\epsilon(\omega, \mathbf{k}_\mathrm{T})}\left[k_{z\mathrm{T}}\hat{\mathbf{x}} - k_x\hat{\mathbf{z}}\right] e^{ik_x x} e^{ik_{z\mathrm{T}}z}, \tag{26}$$

and the transverse component of the material displacement through Eq. 8

$$\mathbf{X}_{\mathrm{T}} = \frac{1}{\omega_{\mathrm{T}}^2 - \omega(\omega + i\gamma)} \left[\beta_{\mathrm{T}}^2 \nabla \times \mathbf{\Theta} + i\omega \frac{\alpha}{\rho} \mathbf{A} \right],$$
$$= \frac{\alpha}{\rho \epsilon_0 \omega} \frac{t_{\mathrm{T}} \left(k_{z\mathrm{T}} \hat{\mathbf{x}} - k_x \hat{\mathbf{z}} \right)}{\omega_{\mathrm{T}}^2 - \omega(\omega + i\gamma)} \left[\frac{1}{\epsilon\left(\omega, \mathbf{k}_{\mathrm{T}}\right)} + \frac{\beta_{\mathrm{T}}^2 \omega^2}{c^2} \frac{1}{\omega_{\mathrm{T}}^2 - \omega\left(\omega + i\gamma\right) - \beta_{\mathrm{T}}^2 k_{\mathrm{T}}^2} \right] e^{ik_x x} e^{ik_{z\mathrm{T}} z}. \tag{27}$$

In the above the out-plane-wavevector for the transverse mode k_{T} is given by the root of Eq. 14 and $\mathbf{k}_{\mathrm{T}} = (k_x, 0, k_{z\mathrm{T}})$. In practice as the wavevector of the transverse mode is small, satisfying $k\beta_{\mathrm{T}} \ll \omega$ it is only necessary to consider this equation in the non-dispersive limit where it reduces to the standard Helmholtz equation.
The electromagnetic fields in the upper halfspace $z > 0$ correspond to a TM polarised plane wave incident from $z = \infty$. They are given by

$$\mathbf{E}_{\mathrm{I}} = \frac{e^{ik_x x}}{\epsilon_0 \epsilon_{\mathrm{B}} \omega} \left[\left(k_{z\mathrm{B}} \hat{\mathbf{x}} - k_x \hat{\mathbf{z}} \right) e^{ik_{z\mathrm{B}} z} - r \left(k_{z\mathrm{B}} \hat{\mathbf{x}} + k_x \hat{\mathbf{z}} \right) e^{-ik_{z\mathrm{B}} z} \right],$$
$$\mathbf{H}_{\mathrm{I}} = \left[e^{ik_{z\mathrm{B}} z} + r e^{-ik_{z\mathrm{B}} z} \right] e^{ik_x x}, \tag{28}$$

where r is the reflection coefficient and k_{B} is the out-of-plane wavevector in the upper halfspace given by

$$k_{z\mathrm{B}} = \sqrt{\epsilon_{\mathrm{B}} \frac{\omega^2}{c^2} - k_x^2}. \tag{29}$$

Boundary Conditions

We start by applying the standard electromagnetic Maxwell boundary conditions. Continuity of the tangential magnetic field yields

$$1 + r = t_{\mathrm{T}}, \tag{30}$$

and that of the tangential electric field gives

$$\frac{k_{z\mathrm{B}}}{\epsilon_{\mathrm{B}}} \left(1 - r\right) = \frac{k_{z\mathrm{T}}}{\epsilon\left(\omega, \mathbf{k}_{\mathrm{T}}\right)} t_{\mathrm{T}} - \frac{2}{\pi} k_{x\mathrm{L}} \frac{\beta_{\mathrm{L}}^2}{c^2} \left(1 - \frac{\epsilon_\infty}{\epsilon\left(\omega, 0\right)} \right) t_{\mathrm{L}}, \tag{31}$$

where we eliminate the homogeneous electric field by considering in addition its counterpart in the upper halfspace as previously described.
Note that, due to Bragg scattering, in applying boundary conditions we have to deal with the mismatch between a fast oscillating longitudinal mode and the slow oscillating transverse ones. In the spirit of the macroscopic approach we employ, we define the longitudinal field averaged over the unit cell

$$\langle \mathbf{E_L} \rangle = \mathbf{E_L}|_{x=0} e^{ik_x x} \int_0^a \frac{\mathrm{dx}}{a} e^{i\frac{\pi}{a} x} = \frac{2i}{\pi} \mathbf{E_L}|_{x=0} e^{ik_x x}, \tag{32}$$

and we utilise this averaged form of the longitudinal field in the calculation of the boundary conditions below. To account for the oscillations of the crystal we also need to apply mechanical boundary conditions. The appropriate choice for a free surface such as that considered here are the continuity of the mechanical forces, or the normal components of the stress tensor. These boundary conditions can be written in the form

$$\frac{\partial X_x}{\partial z} + \frac{\partial X_z}{\partial x} = 0,$$
$$C_{13} \frac{\partial X_x}{\partial x} + C_{33} \frac{\partial X_z}{\partial z} = 0, \tag{33}$$

where C_{13}, C_{33} are elastic coefficients of the lattice. Application of the former boundary condition yields

$$t_{\mathrm{H}} = \frac{1}{k_x^2} \left[-\frac{2}{\pi} \frac{\beta_{\mathrm{L}}^2}{c^2} \frac{\epsilon_\infty}{\epsilon\left(\omega, 0\right)} t_{\mathrm{L}} k_{x\mathrm{L}} k_{z\mathrm{L}} - \frac{t_{\mathrm{T}}}{2} \left(k_{z\mathrm{T}}^2 - k_x^2 \right) \times \left(\frac{\beta_{\mathrm{T}}^2}{c^2} \frac{\omega^2}{\omega_{\mathrm{T}}^2 - \omega\left(\omega + i\gamma\right) - \beta_{\mathrm{T}}^2 k_{\mathrm{T}}^2} + \frac{1}{\epsilon\left(\omega, \mathbf{k}_{\mathrm{T}}\right)} \right) \right], \tag{34}$$

and combining with the latter condition we can find

$$t_{\mathrm{L}} = \frac{\pi}{2i} \frac{t_{\mathrm{T}}}{2} \frac{c^2}{\beta_{\mathrm{L}}^2} \frac{\epsilon\left(\omega, 0\right)}{\epsilon_\infty} \times \frac{\left(C_{33} - C_{13}\right) \left(2ik_x k_{z\mathrm{T}} + \left(k_{z\mathrm{T}}^2 - k_x^2 \right) \right)}{C_{13} k_{x\mathrm{L}}^2 + C_{33} k_{z\mathrm{L}}^2 + i \left(C_{33} - C_{13}\right) k_{x\mathrm{M}} k_{z\mathrm{L}}} \times \left[\frac{\beta_{\mathrm{T}}^2}{c^2} \frac{\omega^2}{\omega_{\mathrm{T}}^2 - \omega\left(\omega + i\gamma\right) - \beta_{\mathrm{T}}^2 k_{\mathrm{T}}^2} + \frac{1}{\epsilon\left(\omega, \mathbf{k}_{\mathrm{T}}\right)} \right]. \tag{35}$$

Finally, combining this result with the electromagnetic boundary conditions we can find a single equation whose solution yields the reflectance coefficient of the halfspace.

$$\frac{k_{z\mathrm{B}}}{\epsilon_{\mathrm{B}}} \left(1 - r\right) = \left(1 + r\right) \left[\frac{k_{z\mathrm{T}}}{\epsilon\left(\omega, \mathbf{k}_{\mathrm{T}}\right)} + \Omega \right], \tag{36}$$

where

$$\Omega = \frac{ik_{x\mathrm{L}}}{2} \left(\frac{\epsilon\left(\omega, 0\right)}{\epsilon_\infty} - 1 \right) \times \frac{\left(C_{33} - C_{13}\right) \left(2ik_x k_{z\mathrm{T}} + k_{z\mathrm{T}}^2 - k_x^2 \right)}{C_{13} k_{x\mathrm{L}}^2 + C_{33} k_{z\mathrm{L}}^2 + i \left(C_{33} - C_{13}\right) k_{x\mathrm{L}} k_{z\mathrm{L}}} \times \left[\frac{\beta_{\mathrm{T}}^2 \omega^2}{c^2} \frac{1}{\omega_{\mathrm{T}}^2 - \omega\left(\omega + i\gamma\right) - \beta_{\mathrm{T}}^2 k_{\mathrm{T}}^2} + \frac{1}{\epsilon\left(\omega, \mathbf{k}_{\mathrm{T}}\right)} \right], \tag{37}$$

leading to the reflection coefficient

$$r = \frac{\frac{k_{z\mathrm{B}}}{\epsilon_{\mathrm{B}}} - \frac{k_{z\mathrm{T}}}{\epsilon(\omega, \mathbf{k}_{\mathrm{T}})} - \Omega}{\frac{k_{z\mathrm{B}}}{\epsilon_{\mathrm{B}}} + \frac{k_{z\mathrm{T}}}{\epsilon(\omega, \mathbf{k}_{\mathrm{T}})} + \Omega}. \tag{38}$$

The result in Eq. (38) has been obtained by considering the equation of motion for an isotropic crystal in Eq. (1),

and then introducing the Bragg scattering of the longitudinal mode along the c-axis. The effect of the Bragg scattering is entirely encoded in Ω and when its value goes to 0 we recover the standard Fresnel coefficient for TM polarised reflection from an isotropic halfspace

$$r^{\mathrm{TM}} = \frac{\frac{k_{z\mathrm{B}}}{\epsilon_{\mathrm{B}}} - \frac{k_{z\mathrm{T}}}{\epsilon(\omega,\mathbf{k}_{\mathrm{T}})}}{\frac{k_{z\mathrm{B}}}{\epsilon_{\mathrm{B}}} + \frac{k_{z\mathrm{T}}}{\epsilon(\omega,\mathbf{k}_{\mathrm{T}})}}. \tag{39}$$

In the case of an uniaxial crystal whose c-axis lies parallel to the x-axis, the effect of the anisotropy can then be reintroduced, to the dominant order, by considering the Fresnel coefficient for a uniaxial crystal, that is by performing in Eqs. (37)-(38) the substitutions

$$k_{z\mathrm{T}}^2 \to \epsilon_{\|}\left(\omega, \mathbf{k}_{\mathrm{T}}\right) \frac{\omega^2}{c^2} - \frac{\epsilon_{\|}\left(\omega, \mathbf{k}_{\mathrm{T}}\right)}{\epsilon_{\perp}\left(\omega, \mathbf{k}_{\mathrm{T}}\right)} k_x^2, \tag{40}$$

$$\epsilon\left(\omega, \mathbf{k}_{\mathrm{T}}\right) \to \epsilon_{\|}\left(\omega, \mathbf{k}_{\mathrm{T}}\right), \tag{41}$$

where $\epsilon_{\|}$ ($\epsilon_{\perp}$) is the dielectric function parallel (perpendicular) to the c-axis.

Supplementary Note 3: Additional Experimental Data

In the main body of the manuscript experimental data was presented from two samples with nominal pillar diameters of 300nm and 500nm. For each nominal pillar diameter 6 unique samples were fabricated. Due to variability in the fabrication procedure the frequencies of the optical modes varies slightly between samples. In Fig. 1 we show the reflectance spectra for the samples omitted from the main body of the text. Note that despite the variation in mode frequencies away from the weak LO phonon, every sample nonetheless reproduces the anticrossing.

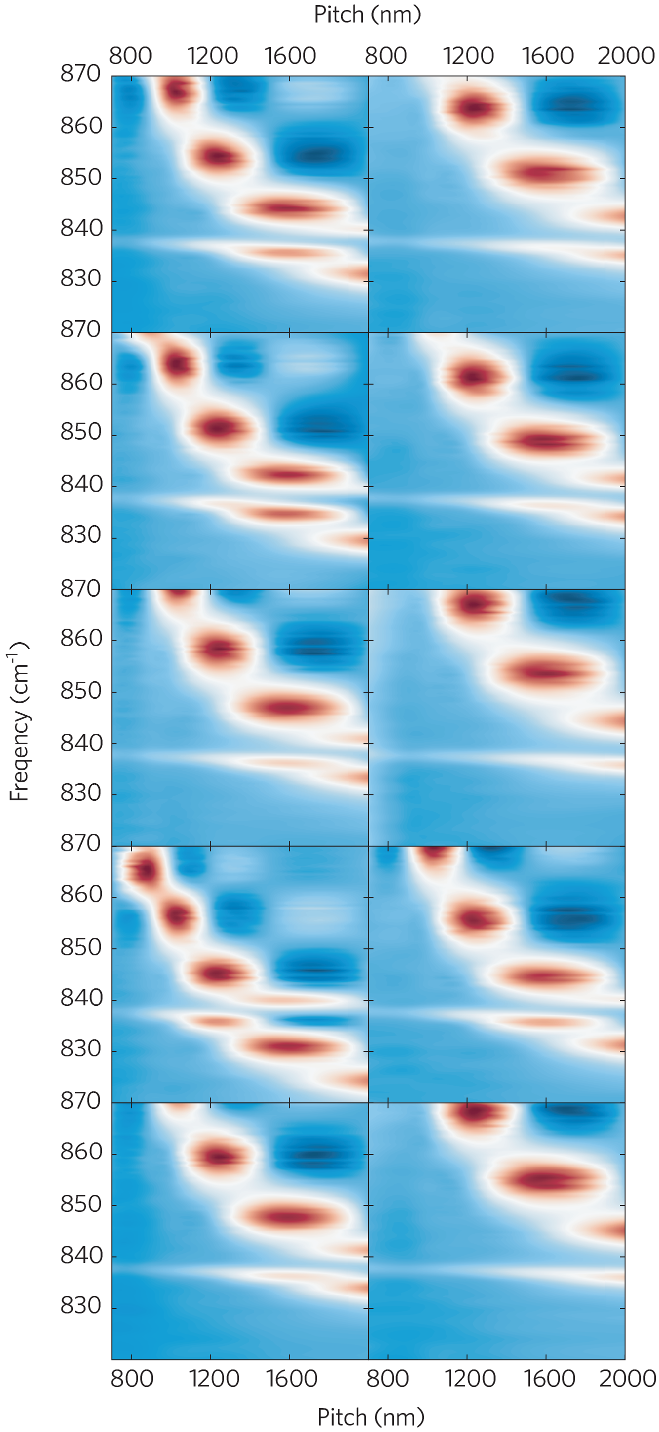

Supplementary Figure 1: Reflectance maps for samples of 300nm (1st Column) and 500nm (2nd Column). The rows correspond to different fabrication runs.

Supplementary References

[1] Trallero-Giner, C., Garcia-Moliner, F., Velasco, V. & Cardona, M. Analysis of the phenomenological models for long-wavelength polar optical modes in semiconductor layered systems. *Physical Review B* **45**, 11944 (1992).

[2] Trallero-Giner, C. & Comas, F. Phenomenological treatment of long-wavelength polar optical oscillations in semiconductor nanostructures. *Philosophical Magazine B* **70**, 583–599 (1994).

[3] Santiago-Pérez, D. G., Trallero-Giner, C., Pérez-Álvarez, R. & Chico, L. Polar optical phonons in core–shell semiconductor nanowires. *Physica E: Low-dimensional Systems and Nanostructures* **56**, 151–159 (2014).
